# How are "teaching the teachers" courses in evidence based medicine evaluated? A systematic review

**DOI:** 10.1186/1472-6920-10-64

**Published:** 2010-09-29

**Authors:** Jacek Walczak, Anna Kaleta, Elżbieta Gabryś, Krzysztof Kloc, Shakila Thangaratinam, Gemma Barnfield, Susanne Weinbrenner, Berit Meyerrose, Theodoros N Arvanitis, Andrea R Horvath, Gianni Zanrei, Regina Kunz, Katja Suter, Bernard Burnand, Chantal Arditi, Katrien Oude Rengerink, Gee Harry, Ben WJ Mol, Khalid S Khan

**Affiliations:** 1 CASPolska, 32-400 Myślenice, ul. Mickiewicza 40, Poland; 2 Birmingham Women's NHS Foundation Trust, Metchley Park Road, Edgbaston, Birmingham B15 2TG, UK; 3 University of Birmingham, Edgbaston, Birmingham B15 2TG, UK; 4 Agency for Quality in Medicine, Wegleystrasse 3, 10623 Berlin, Germany; 5 TUDOR EBM Network, University of Szeged, Albert Szent-Gyorgyi Medical Centre, Semmelweis u. 6, H-6725 Szeged, Hungary; 6 Universita Cattolica del Sacro Curoe, Via Emilia Parmense 84, 29100 Piacenza, Italy; 7 Basel Institute for Clinical Epidemiology and Biostatistics, Hebelstrasse 10, CH 4031 Basel, Switzerland; 8 Academic Medical Center, University of Amsterdam, Department of Obstetrics and Gynaecology, Meibergdreef 9, 1105 AZ Amsterdam, The Netherlands; 9 Centre d'épidémiologie clinique, IUMSP, Bugnon 17, 1005 Lausanne, Switzerland

## Abstract

**Background:**

Teaching of evidence-based medicine (EBM) has become widespread in medical education. Teaching the teachers (TTT) courses address the increased teaching demand and the need to improve effectiveness of EBM teaching. We conducted a systematic review of assessment tools for EBM TTT courses. To summarise and appraise existing assessment methods for teaching the teachers courses in EBM by a systematic review.

**Methods:**

We searched PubMed, BioMed, EmBase, Cochrane and Eric databases without language restrictions and included articles that assessed its participants. Study selection and data extraction were conducted independently by two reviewers.

**Results:**

Of 1230 potentially relevant studies, five papers met the selection criteria. There were no specific assessment tools for evaluating effectiveness of EBM TTT courses. Some of the material available might be useful in initiating the development of such an assessment tool.

**Conclusion:**

There is a need for the development of educationally sound assessment tools for teaching the teachers courses in EBM, without which it would be impossible to ascertain if such courses have the desired effect.

## Background

Evidence-based medicine (EBM), is defined as the integration of the best research evidence with patient's values and clinical circumstances for clinical decision making [1]. However, for EBM to become a reality for practice, studies have shown that it needs to be taught by competent clinical trainers, who help trainees to learn efficiently by exploiting on-the-job learning opportunities [2]. Trainers who have received formal EBM training are more likely to teach application of EBM in a clinical setting [3]. Teaching EBM should not only equip practitioners with knowledge and skills but also foster their attitudes and encourage the practice of EBM. Critical appraisal and EBM teaching that is integrated into clinical practice appears to be effective in improving substantial outcomes, including behavioural changes [4]. One of the objectives of TTT courses should be to bring about a positive change in the teacher's knowledge, skills and attitude towards EBM teaching in a clinical setting. For professional development in this field, many trainers attend teaching the teachers (TTT) courses for EBM, which are widespread. The Leonardo da Vinci programme, part of the European Commission's Lifelong Learning Programme, has funded a EU project involving collaboration of 10 partners within Europe to design an e-learning curriculum for continuing professional development (CPD) on how to teach EBM in a clinical setting http://www.ebm-unity.org[5,6]. Through this project, we have developed a European qualification in Teaching Evidence-Based Medicine which aims to improve transparency across the European healthcare sector [7]. Often teachers assess their own learning through their students' feedback. However, this may be naive as it is well known that there is no direct relationship between feedback and learning achievement [8,9]. The use of assessment tools if any to evaluate such courses is unknown. There are currently many courses that train the trainer in teaching EBM skills. A review of assessment tools used for such courses can help us to gauge how TTT courses are evaluated. We aimed to systematically review existing assessment tools used to evaluate TTT courses in EBM.

## Methods

The systematic review has been conducted by a well designed protocol using robust methodology [10,11] to allow us to identify educationally sound assessments of EBM TTT courses.

We searched the following databases from database inception to November 2008: PubMed, EmBase, BioMed, Cochrane Library and Eric (Education Resources Information Centre, a collection of bibliographic records of education literature). Search strategies for electronic databases were developed based on the review of known relevant literature, and combined MeSH and free text terms, as shown in Additional File 1. We handsearched relevant papers identified through the reference list of retrieved articles, examined our Consortium's personal files, explored for related articles in Pubmed and searched citations in Google Scholar. No language restrictions were applied. Target audiences of the course (teachers) were defined as any health care professional with teaching responsibilities. The definition of a TTT course was based on three characteristics: the aim of the course, the type of participants, and the presence of an assessment strategy. We selected the courses that were related to learning teaching skills in EBM. We included papers that reported development, validation or implementation of any assessment tool or methods to evaluate an EBM TTT course. Due to the specificity of the subject matter we did not use restrictive inclusion criteria related to study quality. All titles and abstracts were screened by two reviewers for studies that met the inclusion criteria. Any disagreement between reviewers was resolved by consensus. We extracted data on the course objectives, duration, target audience, teaching methods, assessment strategy and key outcomes.

## Results

**Figure 1:**
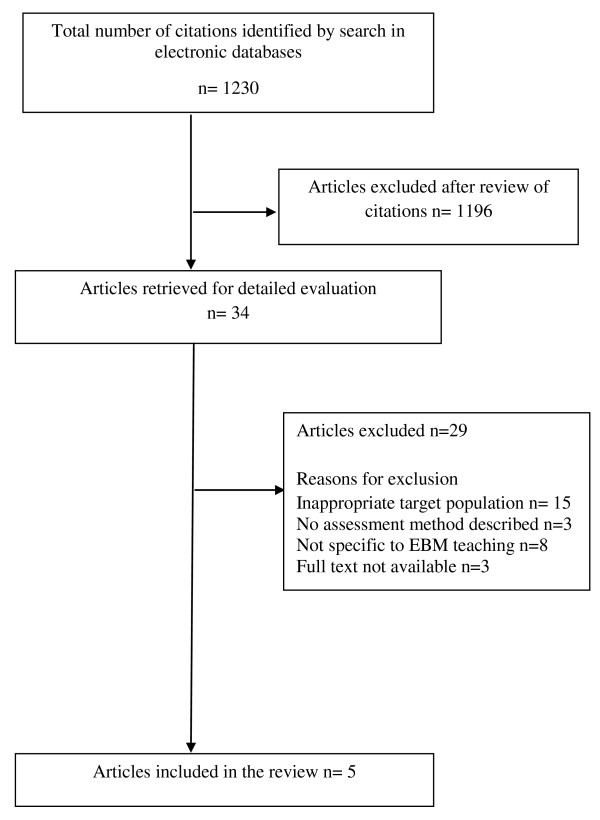
**Flow chart of study selection articles included in the review**.

**O**ur search identified 1230 primary articles, of which five were included in the review (Figure 1). The summary of all TTT courses, which have been included in this systematic review is shown in Additional File 2, providing details of course objectives, target audience, teaching methods, duration of course, assessment methods and key outcomes.

The target audience of the EBM TTT courses identified in the review were faculty members in family practice residences who help residents develop skills in the use of informatics and evidence based medicine [12], podiatric medical educators [13], clinical teachers, and librarians [14-16]. Diverse teaching methods such as instructional workshops, facilitated discussions, hands-on exercises and small group sessions were used in the courses. We did not identify a clinically integrated course teaching application of EBM in routine clinical practice. The total duration of these courses varied from 1 day to 24 months.

The courses used various assessment tools independent of the teaching method to evaluate the effectiveness of the course. The most popular method of course assessment was pre-and post-course appraisal as observed in three of the five chosen articles [12,13,15]. One course surveyed the participants only once, after the completetion of the course. The most detailed assessment method to study the effectiveness of the course was by Scherrer et al [16]. It was a collaboration between librarians and medical faculty to train librarians and health professionals to teach principles of EBM. EBM principles and skills were presented and appropriate teaching methods and techniques were considered. As a method of evaluation, a questionnaire with twenty questions was created and designed to be completed by a twenty-minute telephone interview. After a pilot project, the final survey contained twenty-three questions that took about fifteen minutes to complete. Respondents were asked to state if the course resulted in increased teaching of EBM concepts by the participants and methods used when they returned to their institutions. It explained the influence of the course on the participants in teaching EBM principles, their understanding of EBM principles and confidence in teaching EBM concepts. One of the questions aimed to identify the barriers faced by the participants in teaching EBM. The components of this questionnaire may constitute a basis for the development of an assessment tool to evaluate teaching the teachers courses for EBM [16].

## Discussion

This review has revealed variations in the content of EBM courses, their duration, methods of teaching and course assessment. The reviewers have identified a substantial gap in assessment methods for the evaluation of EBM TTT courses. Very few studies in this field had any detail of course assessments. Existing assessment methods are very few and of poor quality. Moreover the identified assessment tools were intended for teacher' courses focused on courses conducted traditionally (workshops, one-to-one consultation, lectures) rather than clinically integrated courses.

Our review has been the first one to systematically search for assessment tools for teaching the teachers courses in EBM. It was systematically conducted with rigorous methodology and has comprehensively searched, identified and described existing tools for assessing EBM TTT courses. The findings of the review are limited by the small number of published reports on the assessment methods used in this area. EBM can only improve care if it is integrated in clinical practice [17]. This can be achieved by effective application of EBM skills in the clinical environment.To evaluate attainment of this objective teaching the teachers courses need to employ appropriate assessment tools. The TTT EBM curricula is widely based on the model developed in McMaster University, Sackett's books on how to teach EBM and User's guide to medical literature [18]. But little seems to be reported or known about EBM teaching evaluation tools, with much of this knowledge observational [19].

Until now several tools for assessment are known to be used in different kinds of courses: Research Self-Efficacy Scale (RSES), the stages of change (SOC) model and Kirkpatrick's model [9,20,21]. RSES scale has good internal consistency but it has been designed to assess research self-confidence. Thus it lacks face validity for teaching the teachers courses which focus on helping with EBM teaching not on conducting or teaching how to research. The SOC model suffers the same deficiency as it examines learners' attitudes, intentions and actions towards research, not EBM teaching. Moreover, self assessments of this type suffer in the area of criterion validity as there is a loose link between self-perception and objective assessment [22]. The most commonly known tool is the Kirkpatrick's model, which is used frequently as a tool for assessing individual trainee performance but also for measuring the effectiveness of teaching the teacher courses [20,21]. Evaluation of educational interventions must concern at least four dimensions embedded in Kirkpatrick's hierarchy, such as participant's satisfaction, learning (both knowledge and skills), behavioral change (understood as a transfer of knowledge and skills to workplace), and outcomes which are mainly considered as impact on patients [17]. However none of included articles described and made use of this method. Furthermore, change in knowledge may not equate to change in behaviour.

Successful teaching depends not only on good teaching, but also on willingness and effort by the learner, a supportive social environment with teaching and learning opportunities [8,23]. For evaluation of a teacher's performance, can we rely on assessments which utilise trainee's evaluations? Two reasons students' feedback is inappropriate as a measure of teacher quality are that they may not measure all that the teachers are trying to achieve and often do not also provide useful information for teachers about what they need to know to teach more effectively [8,24]. Moreover, data from a randomised controlled trial suggests that trainee feedback can be manipulated by introducing "seduction" in teaching[25]. For these reasons we should evaluate trainers' courses with specifically developed assessment tools. To maintain relevance of the course and to achieve its intended objectives, assessment tools need to be designed as a part of an ongoing evaluation cycle [26].

Effective teacher education is essential to improve EBM teaching quality [8] and constitutes the main reason for development of appropriate assessment tools.

## Conclusion

Due of the lack of well developed assessment tools for courses for EBM teachers, the effect of teaching skills of tutors is currently not measurable. There is a need to develop educationally sound assessment tools to encourage EBM teachers to provide clinically integrated EBM teaching.

## Competing interests

The authors declare that they have no competing interests.

## Authors' contributions

KSK, ST, BWM and JW conceived the project. AK, EG and KK performed literature search, study selection and data extraction. All authors participated in the development of the manuscript and provided input into the final version.

## Funding

The systematic review was prepared within the study which was funded by the European Union Leonardo da Vinci project grant (grant number: UK/07/LLP-LdV/TOI-062).

## Pre-publication history

The pre-publication history for this paper can be accessed here:

http://www.biomedcentral.com/1472-6920/10/64/prepub

## Supplementary Material

Additional file 1 **Search strategy for identification of articles for the review of assessment methods in EBM TTT courses**. Click here for file

Additional file 2 Details of primary articles included in the review of assessment methods of EBM teaching the teachers courses Click here for file
