# Supplementary material for: How are "teaching the teachers" courses in evidence based medicine evaluated? A systematic review
Source: BMC Med Educ. 2010 Sep 29;10:64. doi: 10.1186/1472-6920-10-64 (PMC2958160; doi:10.1186/1472-6920-10-64)
Supplement: Additional file 1 — Search strategy for identification of articles for the review of assessment methods in EBM TTT courses. [file 1472-6920-10-64-S1.DOCX]

**Table 1. Search strategy for identification of articles for the review of assessment methods in EBM TTT courses**

| **Key word category** | **Key words** | **Results** | | | |
| --- | --- | --- | --- | --- | --- |
|  |  | **Pubmed** | **Embase** | **Biomed Central** | **Cochrane Library** |
| **related to learning** | *“learning”* OR *“learn”* OR *“teaching”* OR *“teach”* OR *“training”* OR *“train”* OR *“education”* OR *“educate”* OR *“skill”* OR *“skills”* OR *“knowledge”* OR *“course”* OR *“competence”* | 1 548 779 | 1 168 392 | 31 909 | 70 191 |
|  | AND |  |  |  |  |
| **related to assessment** | *“assessment”* OR *“assessing”* OR *“assess*”* OR *“efficiency”* OR *“efficacy”* OR *“effectiveness”* OR *“effective”* OR *“effectiv*”* OR *“results”* OR *“result”* OR *“result*”* OR *“outcomes”* OR *“outcom*”* OR *“measure”* OR *“measuring”* OR *“measure”* OR *“evaluation”* OR *“evaluate”* OR *“evaluat*”* OR *“perf*or*mance”* OR *“test”* OR *“tests”* OR *“testing”* OR *“test*”* OR *“examination”* OR *“examine”* OR *“examin*”* OR *“implementation”* OR *“implement*”* OR *“tool”* OR *“tools”* OR *“questionary”* OR *“questionnaire”* | 8 839 870 | 8 895 008 | 58 189 | 430 016 |
|  | AND |  |  |  |  |
| **related to EBM** | *“EBM”* OR “*evidence–based”* | 45 626 | 149 146 | 2 635 | 4 791 |
|  | AND |  |  |  |  |
| **related to participants** | *“teacher”* OR*, “teachers”* OR “*trainer”* OR *“trainers”* OR *“tutor”* OR *“tutors”* | 26 372 | 39 618 | 1 201 | 2 048 |
| **Final number** |  | 204 | 441 | 229 | 86 |
