# Supplementary material for: How are "teaching the teachers" courses in evidence based medicine evaluated? A systematic review
Source: BMC Med Educ. 2010 Sep 29;10:64. doi: 10.1186/1472-6920-10-64 (PMC2958160; doi:10.1186/1472-6920-10-64)
Supplement: Additional file 2 — Details of primary articles included in the review of assessment methods of EBM teaching the teachers courses [file 1472-6920-10-64-S2.DOCX]

**Table 2. Details of primary articles included in the review of assessment methods of EBM teaching the teachers courses**

| **Author (year)** | **Target audience** | **Course objectives** | **Teaching methods** | **Course duration** | **Type of assessment** | **Key outcomes** |
| --- | --- | --- | --- | --- | --- | --- |
| Cartwright CA (2002) | Faculty members in family practice residences. | To increase knowledge and skills in the use of information technology in order to help residents develop skills in the use of informatics and evidence-based medicine. | Instructional workshops, EBM mentoring for preceptors. | 24 months (two half-day workshops, mentoring – at least once a month). | The assessment of medical informatics competency and EBM skills before and after the training. | Pre- and post- project assessment demonstrated a significant improvement in computer and EBM skills of faculty members. Informal feedback from residents indicated that these skills have been successfully applied to the practice. |
| Green ML (2005) | Podiatric medical educators. | To help integrate EBM education into the curricula at their home institutions. | Facilitated discussions, minilectures, hands-on exercises, implementation planning, support after the workshop. | 2-day workshop. | Retrospective self-reported ratings before and after the workshop. | Evidence-based medicine practice and teaching skills improved after the train-the trainer faculty-development program. |
| Dorsh JL (2003) | Health and information professionals. | To introduce concepts, methods, and tools of EBM to graduate level students and practicing EBM preceptors. | Lectures, discussions, hands-on computer searching, small group sessions. | One-week course. | Two surveys: Medical Library Association evaluation, University of Illinois at Chicago College of Medicine survey. | Surveys showed that respondents thought, that it was a relevant course, the content was useful and instructors were prepared. |
| Kouhpayehzadeh (2006) | Clinical teachers (physicians). | To improve clinical teachers’ abilities and skills in using EBM. | Group sessions. | Two half-day workshops. | The impact of the workshop on teachers’ attitudes and the use of EBM skills. Pre-course and post-course questionnaires using a 5-point Likert scale. | Attitudes about EBM and self-reported EBM skills improved. |
| Scherrer CS (2006) | Librarians and health professionals. | To learn the basic skills of EBM, share experience how these can be taught and practiced. | A collaboration model between librarians and medical faculty. | One-week course. | The questionnaire comprising 23 questions filled in via a 15-minute telephone interview. Participants were asked if and how they taught EBM on returning to their institution, if they felt competent to critically appraise an article, and if their skills in searching evidence improved. | The TTT model used in this course was reported as an effective approach for promoting and developing the skills which librarians need to teach evidence-based principles. |
